# Housing and Displacement as Risk Factors for Negative Health Outcomes Among People Who Inject Drugs in Los Angeles, CA, and Denver, CO, USA

**DOI:** 10.21203/rs.3.rs-4758949/v1

**Published:** 2024-08-17

**Authors:** Jesse Lloyd Goldshear, Karen F. Corsi, Rachel Carmen Ceasar, Siddhi S. Ganesh, Kelsey A. Simpson, Alex H. Kral, Ricky N. Bluthenthal

## Abstract

Background The United States is currently experiencing a housing and homelessness crisis. In response, many cities have adopted policies of displacement that move unhoused people from place to place. Recent research indicates that these policies may have negative health impacts on unhoused people who use drugs. We sought to examine health risks associated with government-enforced displacement among unhoused people who inject drugs (PWID). Methods We interviewed a community-recruited sample of opioid-using PWID in Los Angeles, CA and Denver, CO between April 2021 and November 2022 (N = 472) about their demographic/socioeconomic characteristics, drug use patterns, housing status, government-enforced displacement including items discarded during displacements, and health risks. We constructed binomial generalized linear regression to examine the risk ratio of non-fatal overdose, and syringe and cooker/cotton sharing between four groups of participants: housed, unhoused and not displaced, unhoused and relocated voluntarily, and unhoused and displaced in the last three months. Results In the last 3 months, 52% of participants were unhoused and displaced by the government. Among those who were displaced, median number of government-enforced displacements was 3 with 69% reporting loss of syringes, 56% loss of naloxone, and 22% loss of buprenorphine medicine. In multivariate models, risk ratios for unhoused and displaced participants were higher for nonfatal overdose and cooker/cotton sharing as compared to housed participants. Risk ratios for syringe sharing amongst unhoused participants did not differ significantly. Conclusions Unhoused and displaced PWID experience elevated health risks. Ending the use of government-enforced displacement of unhoused PWID is essential to reducing health risk in this population.

### Introduction

North America is currently in the midst of two intersecting crises – increasing houselessness and increasing opioid and polysubstance involved overdose deaths.(1) The causes of these twin crises are multifaceted, including the rising cost of housing and growing financial inequality,(2) rising medical costs and other debt,(3) the increasing potency and availability of synthetic opioids like fentanyl,(4) and an increasingly volatile, and unregulated drug supply.(5) In 2022, over 100,000 overdose deaths were reported in the United States.(6) In 2023 the federal Department of Housing and Urban Development (HUD) reported a single-day estimate of 653,104 currently unhoused individuals across the U.S.(7) Among this estimate includes a growing population of people who are *unsheltered*, a status defined by HUD as “residing in a place unfit for human habitation” which includes vehicles, abandoned buildings, and in public areas such as sidewalks and parks.(8) HUD estimated that in 2023, 108,035 (16.5%) unhoused people struggled with “chronic” drug use.(9)

Among unhoused people who use drugs, health risks are often elevated due to a lack of material resources, difficulty accessing hygiene, environmental exposure, and general social abandonment. These health risks include a heightened risk of overdose – fatal and nonfatal – and an increased likelihood of bloodborne infections and skin and soft tissue infections.(10) While many urban and nonurban municipalities have attempted to address these health risks by adopting harm reduction policies,(11) structural interventions to address the growing houselessness contributing to these risk factors appear to be less available.(12)

Instead, policies of forced displacement and ‘urban purification’ have become widely recognized as viable strategies for removing unhoused people from public sight.(13) Commonly referred to as ‘sweeps’, these policies are not new. In cities like Los Angeles, one of the locations of this study, so-called anti-camping and similar ordinances have been utilized for decades to move unhoused individuals from busier and more affluent neighborhoods to Skid Row, an area of concentrated poverty and houselessness in downtown Los Angeles, among other locations.(14) While the use of these government enforced *sweeps* by Los Angeles City was under temporary moratorium during the early stages of the COVID-19 pandemic, their deployment re-emerged in 2021. Other municipalities such as Denver, Colorado, the other location for this study, continued to use sweeps throughout the pandemic. Given the recent US Supreme Court case *City of Grants Pass v. Johnson* – in which justices ruled that polices of displacement were constitutional even when cities do not provide other shelter alternatives – policies of displacement are likely to be even more widely adopted.(15) Often conducted with minimal notice, unhoused community members are forced to rapidly pack up and move their belongings or risk having most of their possessions discarded or confiscated. This is usually done under the threat of arrest. When provided notice, unhoused people who use drugs may also move from an established location voluntarily before a sweep occurs due to hostility from housed neighbors, other changes in the local environment, or wishing to avoid contact with law enforcement.(16) These voluntary moves still represent a source of disruption, and may still include loss of property and disruption of community networks.

For people who use drugs, displacement may represent a distinct source of health risk.(17, 18) A recent simulation study indicated that policies of continually enforced displacement could contribute to thousands of additional deaths among this population, as well as reduce the initiation of medication assisted treatment.(19) Additional recent ethnographic work by members of this team has shown the negative psychological impact of involuntary displacement and confiscation of belongings.(16) In this analysis, we examined if experiences of displacement (government-enforced sweep and voluntary) were associated with health outcomes (non-fatal overdose) and risk behaviors (sharing syringes and cooker/cotton) in Los Angeles and Denver.

## Methods

### Sampling and recruitment

Between April 2021 and November 2022 people who inject drugs (PWID) were recruited from community settings and service organizations that are commonly used by PWID including syringe service sites, public shower, bathroom, and laundry facilities for unhoused people, and other community locations frequented by PWID in Los Angeles, CA Denver, CO. Study eligibility included being 18 years of age or older, self-reported opioid use in the last 30 days, and self-reported drug injection within the past 30 days, which was confirmed by visual inspection of injection sites. After providing informed consent, participants answered questions on demographic and socio-economic characteristics (including unstable housing and displacement), drug use patterns, drug treatment involvement, and other items in a one-on-one computer assisted personal interview using the Questionnaire Development System (Nova Research, Bethesda, MD). Participants received $20 for completing this interview. This cross-sectional analysis includes data from 472 PWID, of which 223 from Los Angeles and 249 from Denver. All study procedures were reviewed and approved by the institutional review board at the University of Southern California.

### Study measures

### Exposure Variables

We were interested in how government-enforced displacement impacted health outcomes and risk among PWID. We created a categorical variable representing both housing status and displacement type. For participants who reported being unhoused, spending at least one night in the last three months in a tent, outdoors, vehicle, abandon building/garage/shed, and who reported at least one move in that time, we asked, “In the last 3 months, how many of these moves where because of police, security guards, or other city/county officials?” Participants reporting one or more government-involved moves were classified as “unhoused and displaced by the government.” Participants reporting at least one move but zero due to the government were classified as “unhoused and moved”. Those reporting being unhoused and not moving for any reason were classified as “unhoused and not moved.” The remaining participants were classified as “housed.”

Participants were also asked if any of the following items had been discarded by “city, state, or county employees such as police or sanitation workers?” in the past three months. The item listed included identification, other important paperwork, Naloxone/Narcan, Suboxone/Subutex (buprenorphine), chronic condition medication (e.g., diabetes), acute condition medication (e.g. bacterial infection), clothing, food, illicit drugs, photos, pets, syringes, cotton or cookers (paraphernalia), biohazard containers, and tents or shelter. This list included material that is important for reducing fatal overdose risk, HIV and HCV transmission risk, and for maintaining opioid treatment and withdrawal management.

## Outcome Variables

Outcome variables for this analysis included nonfatal overdose, receptive syringe sharing, and sharing cotton or cookers. Nonfatal overdose was assessed as an affirmative answer to: “In the last 3 months, have you overdosed?” Participants who reported one or more instances of injecting ”…using syringes/needles that you know had been used by someone else (including a close friend or lover)?” were classified as having shared syringes. Participants who responded yes to having “…shared a cooker, spoon, or shaker that someone else used before you?” and/or “…have used a filter or cotton that someone else used before you?” were classified as having shared a cooker or cotton.

## Potentially Confounding Variables

We considered the following demographic and socioeconomic variables as potential covariates in this study: gender (male or not male), race/ethnicity (white, Black, Latinx, Native American, Asian or Pacific Islander, mixed), age (< 30, 30 to 39, 40 to 49, or 50 or older), relationship status (single, married, living as married), sexual partners (types – steady, casual, paid), and sexual orientation (heterosexual, bisexual, gay, or lesbian), monthly income (<$1,000, $1,001 to $1,400, or 1,401 to $2,100, or $2,101 or more), income sources (job, unemployment, veteran’s benefits, welfare, disability, social security, spouse, family, friends, recycling, panhandling, and illegal or possibly illegal sources), and education (high school graduate or equivalent).

Drug use measures included injection frequency, types and times of drugs used by route (injection or non-injection), and number of years since first injection. We converted injection frequency in the last three months into a categorical variable with the following classifications: less than daily use (< 89 injections), at least once but less than three times a day (90 to 269 injections), and three or more times a day ( $\geq$ 270 injections). Number of years since first injection was categorized as < 10 years, 10 to 19 years, and 20 or more years. We also considered factors that have been associated with health outcome variables including contact with security guards, police and other criminal justice involvement (e.g. parole and probation),(20, 21) receiving and giving assistance with injection, syringe coverage and syringe reuse,(22, 23) withdrawal symptoms from opioids and methamphetamine,(24, 25) rushed injection,(26, 27) enrollment in substance use treatment (e.g. current enrollment and enrollment in the last three months in methadone, buprenorphine, residential, and outpatient treatment), and any mental health disorder diagnosis.

## Statistical analysis

After descriptive analyses, we conducted bivariate analyses to determine factors associated at the p < 0.05 level with nonfatal overdose, receptive syringe sharing, and sharing cooker or cotton using chi-square tests for categorical variables and t-tests for continuous variables. We considered the variables listed above in the domains of demographics, socioeconomic characteristics, displacement, and substance use behaviors as potential correlates and confounders. Variables significant in bivariate analyses were then assessed for collinearity within domains using Pearson correlation coefficients. Collinear variables (Pearson correlation coefficient > 0.30) were included in the final analyses as confounders if they were also significantly associated with the displacement exposure variable.

We used binomial generalized linear regression to calculate risk ratios (RR) for the outcome variables while controlling for confounding.(28, 29) Variables not significant in multivariate analyses ($p > 0.05$) at each step were removed from models with the exception of recruitment city. We included recruitment city in all final multivariable models due to statistically significant differences in government-enforced displacement by city ($p < 0.001$). For models, RR and 95% Confidence Intervals (CI) were estimated. All analyses were performed using SPSS, Version 25.(30)

### Results

Our sample consisted of 472 participants (249 from Denver and 223 from Los Angeles), of which 77% were male, 52% were White, 26% were Latinx, 10% were Native American, and 6% were Black (Table 1). Participants were between the ages of 30 and 39 (35%), followed by 25% who were over 50 years old, and 24% who were between 40 and 49 years old. The majority were unstably housed (84%), high school educated (77%), single (72%), and low income (53% had monthly income of less than $1,000 USD). In terms of drug use, 86% reported daily opioid use (including heroin, fentanyl, and/or prescription opioids), 70% reported daily injection, and 59% reported daily methamphetamine use.

| Table 1: Selected demographic, socioeconomic, drug use and health characteristics of opioid-using people who inject drugs in Denver, CO and Los Angeles, CA, 2021/22 (N=472). | | | | | |
|---|---|---|---|---|---|
| **Characteristics** | **Total** N=472 % | **Housed** N=77 % | **Unhoused not moved** n=99 % | **Unhoused, moved** n=51 % | **Unhoused, displaced by government** n=245 % |
| **Site*** | | | | | |
| Denver | 53% | 27% | 34% | 43% | 70% |
| Los Angeles | 47% | 73% | 66% | 57% | 30% |
| **Gender** | | | | | |
| Male | 77% | 68% | 80% | 75% | 80% |
| **Race*** | | | | | |
| White | 52% | 44% | 42% | 45% | 60% |
| Latinx | 26% | 38% | 37% | 31% | 17% |
| African American | 6% | 7% | 8% | 4% | 4% |
| Asian/Pacific Islander | 1% | 0% | 0% | 0% | 2% |
| Native American | 10% | 5% | 4% | 16% | 13% |
| Mixed Race | 5% | 6% | 8% | 4% | 4% |
| **Age (years)*** | | | | | |
| <30 | 16% | 9% | 11% | 21% | 19% |
| 30-39 | 35% | 21% | 27% | 39% | 41% |
| 40-49 | 24% | 26% | 26% | 20% | 24% |
| ≥50 | 25% | 44% | 35% | 20% | 16% |
| **High school education or more** | 77% | 75% | 72% | 75% | 81% |
| **Monthly Income** | | | | | |
| < $1,000 | 53% | 47% | 51% | 66% | 53% |
| $1,001 - $1,400 | 19% | 23% | 18% | 14% | 18% |
| $1,401 - $2,100 | 13% | 10% | 18% | 8% | 14% |
| > $2,101 | 15% | 20% | 13% | 12% | 15% |
| **Drug use, last 3 months** | | | | | |

| | | | | | |
|---|---|---|---|---|---|
| Crack cocaine* | 33% | 20% | 25% | 45% | 38% |
| Powder cocaine | 31% | 22% | 29% | 26% | 35% |
| Methamphetamine* | 84% | 62% | 82% | 82% | 91% |
| Heroin | 82% | 88% | 82% | 75% | 82% |
| Fentanyl* | 70% | 40% | 65% | 69% | 81% |
| Speedball* | 29% | 21% | 21% | 22% | 36% |
| Goofball* | 63% | 42% | 58% | 59% | 74% |
| Cannabis* | 75% | 66% | 70% | 73% | 81% |
| **Non-prescription use** | | | | | |
| Opioids | 28% | 22% | 25% | 22% | 33% |
| Tranquilizers* | 38% | 26% | 31% | 37% | 44% |
| Stimulants | 13% | 8% | 11% | 10% | 15% |
| Methadone | 16% | 17% | 8% | 14% | 18% |
| Buprenorphine* | 14% | 8% | 7% | 14% | 18% |
| **Rushed injection, last 3 months*** | | | | | |
| None | 36% | 52% | 39% | 43% | 29% |
| 1 to 9 times | 29% | 27% | 29% | 29% | 29% |
| 10 times or more | 35% | 21% | 31% | 28% | 42% |
| **Gave an injection to another PWID in the last 3 months*** | 55% | 25% | 54% | 53% | 66% |
| **Received an injection from another PWID, last 3 months** | 34% | 25% | 30% | 28% | 39% |
| **Any syringe reuse, last 3 months** | 40% | 30% | 33% | 45% | 44% |
| **Criminal legal contact, last 3 months** | | | | | |
| Police* | 44% | 16% | 35% | 37% | 57% |
| Parole | 5% | 1% | 6% | 6% | 5% |
| Probation | 10% | 5% | 8% | 8% | 13% |
| Arrest* | 12% | 0% | 13% | 10% | 16% |
| Security guard* | | | | | |

| | | | | | |
|---|---|---|---|---|---|
| | 47% | 23% | 42% | 31% | 60% |
| **Nonfatal overdose, last 3 months*** | 21% | 7% | 19% | 16% | 28% |
| **Receptive syringe sharing, last 3 months*** | 21% | 5% | 18% | 37% | 25% |
| **Cooker/Cotton sharing, last 3 months*** | 41% | 14% | 36% | 45% | 49% |
| * p = <0.05 | | | | | |

In the last three months, 16% (77/472) of participants were housed, 21% (99/472) were unhoused but indicated no displacement, 11% (51/472) were unhoused and moved voluntarily, 52% (245/472) were unhoused and forcibly displaced by government action. For those people who were unhoused, the mean nights outdoors in the last 3 months was 58.23 (standard deviation [STD]=33.65; median=70, interquartile range [IQR]=25, 90). Among those who moved at least once for any reason in the past three months, the median number of moves was 4 (IQR=1, 20). The median number of moves due to government displacement was 3 (IQR=1,10).

Over half (64% or 157/245) of participants who were displaced by the government reported having items taken by government officials (mean number of items taken 7.56, STD=3.54=2; median=8; IQR=5,10) with clothing being the most commonly taken item (93%). Among those who were displaced and had items taken, reports of having health-related items taken at least once in the last three months included syringes (73%) and cookers/cotton (68%) for HIV/HCV prevention, naloxone (60%) for overdose reversal, and buprenorphine (26%) for opioid use disorder (**Figure 1**). Participants reporting government displacement reported higher prevalence of having any items discarded (p<0.05) than participants who were unhoused but not displaced. In chi-square and Fisher exact tests, there were significant differences in prevalence of specific items discarded (paperwork, naloxone, suboxone/buprenorphine, food, photos, biohazard containers, and tents) and displacement category among unhoused individuals. For those items which were significantly different, we produced bivariate models to determine specific risk ratios between item confiscation and displacement category (**Table 2**). Among homeless participants who had items taken, those who were subject to government displacement had significantly (p < 0.05) increased risk of losing all items listed above except naloxone. We found no statistically significant bivariate associations between reporting having items discarded and any of our three model outcome variables.

| Table 2: Binomial generalized linear models of displacement category on items discarded in the last 3 months among unhoused people who inject drugs in Denver and Los Angeles (n = 209) | | | |
|---|---|---|---|
| **Item Taken** | **No moves** | **Voluntary moves** | **Government Displaced** |
| | ARR (95% CI) | ARR (95% CI) | ARR (95% CI) |
| Paperwork | Referent | 1.12 (0.64, 1.97) | 1.49 (1.04, 2.14)* |
| Naloxone | Referent | 0.79 (0.38, 1.66) | 1.37 (0.93, 2.02) |
| Suboxone/Buprenorphine | Referent | 3.18 (0.58, 17.28) | 4.62 (1.17, 18.22)* |
| Food | Referent | 1.11 (0.71, 1.73) | 1.40 (1.05, 1.86)* |
| Photos | Referent | 1.06 (0.37, 3.03) | 2.08 (1.10, 3.92)* |
| Biohazard containers | Referent | 1.16 (0.51, 2.60) | 1.95 (1.17, 3.25)* |
| Tents | Referent | 1.35 (0.64, 2.86) | 2.35 (1.42, 3.88)* |

After adjusting for recruitment site and other confounding variables, being unhoused and not displaced significantly increased risk of nonfatal overdose (RR=2.71, 95% Confidence Interval [CI]=1.07, 6.82, p=0.03), as did being unhoused and displaced by government action (RR=3.84, 95% CI=1.60, 9.20, p=0.002) when compared to housed participants (**Table 3**).

| Table 3: Binomial generalized linear model of nonfatal overdose in the last 3 months among people who inject drugs in Denver and Los Angeles (N=472) | | | |
|---|---|---|---|
| **Factor** | **Adjusted Risk Ratio** | **95% confidence interval** | **p-value** |
| **Housing status** | | | |
| Housed | Referent | - | - |
| Unhoused, not moved | 2.70 | 1.07, 6.82 | 0.035 |
| Unhoused, moved | 2.52 | 0.89, 7.14 | 0.082 |
| Unhoused, government displaced | 3.84 | 1.60, 9.20 | 0.002 |
| **Rushed injection in the last 3 months** | | | |
| Not rushed | Referent | - | - |
| Rushed 1 to 9 times | 1.81 | 1.11, 2.99 | 0.018 |
| Rushed 10 or more times | 2.04 | 1.26, 3.28 | 0.003 |
| **Injected by another person in the last 3 months** | | | |
| No | Referent | - | - |
| Yes | 1.70 | 1.21, 2.37 | 0.002 |
| **Recruitment Location** | | | |
| Denver | Referent | - | - |
| Los Angeles | 1.39 | 0.99, 1.95 | 0.058 |

In a multivariate model that adjusted for confounding variables, including recruitment site (**Table 4**), being unhoused and voluntarily moving (RR=4.54, 95% CI=1.68, 12.27, p=0.003) and being displaced (RR=2.76, 95% CI=1.04, 7.29, p = 0.041) was significantly associated with increased risk of receptive syringe sharing as compared to housed participants.

| Table 4: Binomial generalized linear model of receptive syringe sharing in the last 3 months among people who inject drugs in Denver and Los Angeles (n=472). | | | |
|---|---|---|---|
| **Factor** | **Adjusted Risk Ratio** | **95% confidence interval** | **p-value** |
| **Housing status** | | | |
| Housed | Referent | - | - |
| Unhoused, not moved | 2.75 | 1.00, 7.56 | 0.051 |
| Unhoused, moved | 4.54 | 1.68, 12.27 | 0.003 |
| Unhoused, government displaced | 2.76 | 1.04, 7.29 | 0.041 |
| **Rushed injection in the last 3 months** | | | |
| Not rushed | Referent | - | - |
| Rushed 1 to 9 times | 1.45 | 0.86, 2.45 | 0.163 |
| Rushed 10 or more times | 2.09 | 1.32, 3.38 | 0.002 |
| **Any syringe reuse** | | | |
| No | Referent | - | - |
| Yes | 2.06 | 1.44, 2.94 | <0.001 |
| **Any goofball use in the last 3 months** | | | |
| No | Referent | - | - |
| Yes | 1.78 | 1.13, 2.81 | 0.014 |
| **Recruitment Location** | | | |
| Denver | Referent | - | - |
| Los Angeles | 0.79 | 0.53, 1.18 | 0.251 |

In a multivariate model that adjusted for confounding variables, including recruitment site (**Table 5**), all categories of being unhoused significantly increased risk of sharing either cookers/cottons (unhoused and not moved/displaced: RR=2.26, 95% CI=1.25, 4.06, p=0.007; unhoused and moved voluntarily: RR=2.59, 95% CI=1.41, 4.75, p=0.002 and being unhoused and displaced by government: RR=2.46, 95% CI=1.40, 4.32, p=0.002) as compared to housed participants.

| Table 5: Binomial generalized linear model of cooker/cotton sharing in the last 3 months among people who inject drugs in Denver and Los Angeles (N=472). | | | |
|---|---|---|---|
| **Factor** | **Adjusted Risk Ratio** | **95% confidence interval** | **p-value** |
| **Housing status** | | | |
| Housed | Referent | | |
| Unhoused, not moved | 2.26 | 1.25, 4.06 | 0.006 |
| Unhoused, moved | 2.59 | 1.41, 4.75 | 0.002 |
| Unhoused, government displaced | 2.46 | 1.40, 4.33 | 0.002 |
| **Rushed injection in the last 3 months** | | | |
| Not rushed | | | |
| Rushed 1 to 9 times | Referent | | |
| Rushed 10 or more times | 1.43 | 1.07, 1.91 | 0.017 |
| | 1.56 | 1.18, 2.06 | 0.002 |
| **Any goofball use in the last 3 months** | | | |
| No | | | |
| Yes | Referent | | |
| | 1.75 | 1.31, 2.35 | < 0.001 |
| **Any opioid withdrawal symptoms in the last 3 months** | | | |
| No | | | |
| | Referent | | |
| Yes | | | |
| | 1.98 | 1.34, 2.89 | < 0.001 |
| **Recruitment Site** | | | |
| Denver | Referent | | |
| Los Angeles | 1.06 | 0.87, 1.30 | 0.562 |

## Discussion

Results of this study indicate that among communities of PWID in Los Angeles, California and Denver, Colorado, being unhoused, even when not subject to displacement, was associated with negative health risk outcomes including nonfatal overdose, receptive syringe sharing, and sharing injection-related materials. In several of our models government-enforced displacement of unhoused PWID was also associated with some of these same outcomes. Confiscation and/or disposal of material possessions among our participant population was high and represents an added layer of vulnerability and potential health risk. These results highlight the risk posed by the growing criminalization of homelessness – and specifically of homeless PWID – in the US and are especially relevant in light of the *City of Grants Pass v. Johnson* decision.(15)

The prevalence of having material items confiscated and discarded among our participants is of concern given the extreme health vulnerability of homeless PWID. The increased risk experienced by displaced participants of losing items such as medications for opioid use disorder, biohazard containers for syringe storage, and tents/shelter materials indicates that government displacement is an inherently resource-depriving policy beyond the searches and seizures that homeless communities already experience under so-called "quality of life" laws.(31) While we were not able to include these variables in our main health outcome models due to variable structure and sample size constraints, we believe that the loss of these and other items creates further health vulnerability among this already socially and economically marginalized population. The increased risk of confiscation of medication, syringe storage containers, and basic shelter leaves homeless PWID more vulnerable to potential overdose, bloodborne infection risk, and skin and soft tissue infections.

The elevated risk of nonfatal overdose in association with government-enforced displacement may be explained similarly. It is possible that these displacement events – and the complaint-oriented policing that often precedes them (32) - broke up local drug markets and moved participants into differing, less familiar drug markets and use environments. In this way, voluntary and government-enforced displacement events may produce similar outcomes to direct policing of drug markets.(33–35) These unfamiliar markets may have presented participants with drugs of unknown quality and potency (including unwanted fentanyl), as well as novel adulterants that could have heightened overdose potential. As these overdoses were non-fatal, we do not believe there was an unmeasured relationship with naloxone access, especially given that displacement was not associated with increased risk of naloxone confiscation.

Similarly, we do not believe there to be an unmeasured association between syringe and injection supply access via syringe service programs (SSPs), syringe sharing, and government displacement. The majority (88%) of participants obtained syringes from an SSP in the past three months, and many of them were recruited at or near an SSP. Given the distribution and range of syringe service programs (SSP) and other harm reduction services in Los Angeles and Denver, we assumed that displacement may have moved unhoused people who use drugs further away from these services. Multiple studies have shown that distance and travel time to these programs are predictors of health risk behaviors among PWID.(36, 37) However, in our sample we did not observe a significant association between past three month SSP utilization and movement or displacement.

The results of our analysis should be interpreted with several potential limitations in mind. As data presented here are cross-sectional, no causal relationships could be assessed. Several forms of bias inherent to survey-based research may also have influenced collected data, including participant recall and social desirability bias, although measures similar to the ones used in this study have been found to be reliable and valid in other samples of people who inject drugs.(38, 39) Additionally, while the data collected was comprehensive with hundreds of survey items presented to participants, there are potentially unaccounted for confounders that may have influenced the magnitude of statistical associations. As the majority of our participants were recruited at or near SSPs, our data may have been biased towards those PWID who were able to access an SSP regardless of displacement experience. Future work examining displacement and resource access should aim to recruit a more geographically diverse sample.

## Conclusions

These results are situated within a large body of literature examining the multitude of risk environment characteristics that influence drug use behaviors and health outcomes. Our analysis indicates that displacement and communal breakup of unhoused people who use drugs, whether forced or voluntarily, may influence health risk outcomes. As the number of unhoused individuals in the United States continues to grow, we believe that the implementation of policies of forcible and coerced displacement will contribute to increases in associated negative health outcomes. This potentially sets back much of the progress made by the success of harm reduction programming on overdose and new HIV and Hepatitis C infections, in addition to worsening the conditions that exacerbate skin and soft tissue infections.(19) Based on the outcomes of this emerging body of research, we encourage policymakers and governments to halt policies of forced displacement, and instead adopt alternative strategies including low-barrier, housing first models, and expanded mental health and substance use disorder treatment.(40)

## Abbreviations

HUD – Housing and Urban Development

PWID – People who inject drugs

RR – Risk Ratio

STD – Standard deviation

IQR – Interquartile range

CI – Confidence interval

SSP – Syringe services program

## Declarations

*Ethics approval and consent to participate*

All study procedures were reviewed and approved by the institutional review board at the University of Southern California.

*Consent for publication*

Not Applicable.

*Availability of data and materials*

The datasets generated and/or analyzed during the current study are not publicly available due to contents which could potentially identify research participants engaged in illegal activity, but are available from the corresponding author [JLG] and last author [RNB] on reasonable request.

*Competing interests*

The authors declare that they have no competing interests.

*Funding*

Funding: NIH/NIDA grants #R01DA046049, F31DA054763, T32DA023356.

*Authors' contributions*

JLG wrote main manuscript text, conducted analysis, and acquired funding.

RNB conducted analysis, reviewed manuscript, and acquired funding.

KFC reviewed manuscript and acquired funding.

RCC provided conceptual input for analysis and reviewed manuscript.

SSG helped prepare tables and reviewed manuscript.

KAS reviewed manuscript.

AHK reviewed manuscript.

*Acknowledgements*

We would like to thank our participants for taking the time to sit down and be a part of this study. It would not have been possible without their willingness and candor. We would also like to thank for research coordinators Karina Dominguez Gonzalez and Cheyenne Page, and field staff including Eric Kovalsky and Patricia Wilkins (Denver), Gilbert Portillo, Anthony DiMario, Pooja Shah, Srehith Sannaredy, Andrew Bong, and Sarayu Jaladanki (Los Angeles) for approaching, consenting, enrolling, and interviewing participants.

## References

1. Aronowitz SV, Engel-Rebitzer E, Lowenstein M, Meisel Z, Anderson E, South E. “We have to be uncomfortable and creative”: Reflections on the impacts of the COVID-19 pandemic on overdose prevention, harm reduction & homelessness advocacy in Philadelphia. SSM - Qual Res Health. 2021 Dec 1;1:100013.
2. Kushel M. Homelessness among older adults: An emerging crisis. Generations [Internet]. 2020 Jun 1 [cited 2023 Jul 1];44(2). Available from: https://escholarship.org/uc/item/5601g730
3. Bielenberg JE, Futrell M, Stover B, Hagopian A. Presence of Any Medical Debt Associated With Two Additional Years of Homelessness in a Seattle Sample. Inq J Health Care Organ Provis Financ. 2020 Jan 1;57:0046958020923535.
4. Ciccarone D. The Rise of Illicit Fentanyls, Stimulants and the Fourth Wave of the Opioid Overdose Crisis. Curr Opin Psychiatry. 2021 Jul 1;34(4):344–50.
5. Singh VM, Browne T, Montgomery J. The Emerging Role of Toxic Adulterants in Street Drugs in the US Illicit Opioid Crisis. Public Health Rep. 2020 Jan 1;135(1):6–10.
6. Provisional Data Shows U.S. Drug Overdose Deaths Top 100,000 in 2022 | Blogs | CDC [Internet]. 2023 [cited 2023 Jul 1]. Available from: https://blogs.cdc.gov/nchs/2023/05/18/7365/
7. de Sousa T, Andrichik A, Prestera E, Rush K, Tano C, Wheeler M, et al. The 2023 Anual Homelessness Assessment Report (AHAR) to Congress: Part 1 - Point-in-Time Estimates of Homelessness [Internet]. The U.S. Department of Housing and Urban Development; 2023 Dec. Available from: https://www.huduser.gov/portal/datasets/ahar/2023-ahar-part-1-pit-estimates-of-homelessness-in-the-us.html
8. Homeless Emergency Assistance and Rapid Transition to Housing (HEARTH): Defining Homeless Final Rule [Internet]. [cited 2023 Jul 1]. Available from: https://www.hudexchange.info/resource/1928/hearth-defining-homeless-final-rule
9. Continuum of Care Homeless Assistance Programs Homeless Populations and Subpopulations. United States Department of Housing and Urban Development; 2023 Nov.
10. Ceasar RC, Goldshear JL, Brothers S, Wenger LD, Kral AH, Bluthenthal RN. Factors associated with injury and blood-borne infection risk when providing assisted injection among people who inject drugs. Int J Drug Policy. 2021 Nov;97:103297.
11. Klein A. Harm Reduction Works: Evidence and Inclusion in Drug Policy and Advocacy. Health Care Anal. 2020 Dec 1;28(4):404–14.
12. Doran KM, Fockele CE, Maguire M. Overdose and Homelessness—Why We Need to Talk About Housing. JAMA Netw Open. 2022 Jan 7;5(1):e2142685.
13. Herring C, Yarbrough D, Marie Alatorre L. Pervasive Penality: How the Criminalization of Poverty Perpetuates Homelessness. Soc Probl. 2020 Feb 1;67(1):131–49.
14. Gudis C. Containment and Community: The History of Skid Row and its Role in the Downtown Community Plan [White Paper]. 2022.

15. City of Grants Pass v. Johnson. 2024.
16. Goldshear JL, Kitonga N, Angelo N, Cowan A, Henwood BF, Bluthenthal RN. “Notice of major cleaning”: A qualitative study of the negative impact of encampment sweeps on the ontological security of unhoused people who use drugs. Soc Sci Med. 2023 Dec 1;339:116408.
17. Chiang JC, Bluthenthal RN, Wenger LD, Auerswald CL, Henwood BF, Kral AH. Health risk associated with residential relocation among people who inject drugs in Los Angeles and San Francisco, CA: a cross sectional study. BMC Public Health. 2022 Apr 25;22(1):823.
18. Chang JS, Riley PB, Aguirre RJ, Lin K, Corwin M, Nelson N, et al. Harms of encampment abatements on the health of unhoused people. SSM - Qual Res Health. 2022 Dec 1;2:100064.
19. Barocas JA, Nall SK, Axelrath S, Pladsen C, Boyer A, Kral AH, et al. Population-Level Health Effects of Involuntary Displacement of People Experiencing Unsheltered Homelessness Who Inject Drugs in US Cities. JAMA. 2023 May 2;329(17):1478–86.
20. Bluthenthal RN, Kral AH, Erringer EA, Edlin BR. Drug Paraphernalia Laws and Injection-Related Infectious Disease Risk among Drug Injectors. J Drug Issues. 1999 Jan;29(1):1–16.
21. Markwick N, McNeil R, Small W, Kerr T. Exploring the Public Health Impacts of Private Security Guards on People Who Use Drugs: a Qualitative Study. J Urban Health. 2015 Dec 1;92(6):1117–30.
22. Bluthenthal RN, Anderson R, Flynn NM, Kral AH. Higher syringe coverage is associated with lower odds of HIV risk and does not increase unsafe syringe disposal among syringe exchange program clients. Drug Alcohol Depend. 2007 Jul 10;89(2):214–22.
23. Kral AH, Anderson R, Flynn NM, Bluthenthal RN. Injection Risk Behaviors Among Clients of Syringe Exchange Programs With Different Syringe Dispensation Policies. JAIDS J Acquir Immune Defic Syndr. 2004 Oct 1;37(2):1307.
24. Bluthenthal RN, Simpson K, Ceasar RC, Zhao J, Wenger L, Kral AH. Opioid withdrawal symptoms, frequency, and pain characteristics as correlates of health risk among people who inject drugs. Drug Alcohol Depend. 2020 Jun 1;211:107932.
25. Zhao J, Kral AH, Simpson KA, Ceasar RC, Wenger LD, Kirkpatrick M, et al. Factors associated with methamphetamine withdrawal symptoms among people who inject drugs. Drug Alcohol Depend. 2021 Jun 1;223:108702.
26. Bazazi AR, Zelenev A, Fu JJ, Yee I, Kamarulzaman A, Altice FL. High prevalence of non-fatal overdose among people who inject drugs in Malaysia: Correlates of overdose and implications for overdose prevention from a cross-sectional study. Int J Drug Policy. 2015 Jul 1;26(7):675–81.
27. Ti L, Hayashi K, Kaplan K, Suwannawong P, Wood E, Kerr T. Contextual Factors Associated with Rushed Injecting Among People Who Inject Drugs in Thailand. Prev Sci. 2015 Feb 1;16(2):313–20.
28. WACHOLDER S. BINOMIAL REGRESSION IN GLIM: ESTIMATING RISK RATIOS AND RISK DIFFERENCES1. Am J Epidemiol. 1986 Jan 1;123(1):174–84.
29. Diaz-Quijano FA. A simple method for estimating relative risk using logistic regression. BMC Med Res Methodol. 2012 Feb 15;12(1):14.

30. IBM SPSS Statistics for Windows. Armonk, NY: IBM Corp.; 2017.
31. Robinson T. No Right to Rest: Police Enforcement Patterns and Quality of Life Consequences of the Criminalization of Homelessness. Urban Aff Rev. 2019 Jan 1;55(1):41–73.
32. Herring C. Complaint-Oriented Policing: Regulating Homelessness in Public Space. Am Sociol Rev. 2019 Oct 1;84(5):769–800.
33. Kerr T, Small W, Wood E. The public health and social impacts of drug market enforcement: A review of the evidence. Int J Drug Policy. 2005 Aug 1;16(4):210–20.
34. Wood E, Spittal PM, Small W, Kerr T, Li K, Hogg RS, et al. Displacement of Canada's largest public illicit drug market in response to a police crackdown. CMAJ. 2004 May 11;170(10):1551–6.
35. Ray B, Korzeniewski SJ, Mohler G, Carroll JJ, del Pozo B, Victor G, et al. Spatiotemporal Analysis Exploring the Effect of Law Enforcement Drug Market Disruptions on Overdose, Indianapolis, Indiana, 2020–2021. Am J Public Health. 2023 Jul;113(7):750–8.
36. Whiteman A, Burnett J, Handanagic S, Wejnert C, Broz D. Distance matters: The association of proximity to syringe services programs with sharing of syringes and injecting equipment - 17 U.S. cities, 2015. Int J Drug Policy. 2020 Nov 1;85:102923.
37. Williams CT, Metzger DS. Race and Distance Effects on Regular Syringe Exchange Program Use and Injection Risks: A Geobehavioral Analysis. Am J Public Health. 2010 Jun;100(6):1068–74.
38. Weatherby NL, Needle R, Cesari H, Booth R, McCoy CB, Watters JK, et al. Validity of self-reported drug use among injection drug users and crack cocaine users recruited through street outreach. Eval Program Plann. 1994 Oct 1;17(4):347–55.
39. Needle R, Fisher DG, Weatherby N, Chitwood D, Brown B, Cesari H, et al. Reliability of self-reported HIV risk behaviors of drug users. Psychol Addict Behav. 1995;9(4):242–50.
40. Padgett DK, Stanhope V, Henwood BF, Stefancic A. Substance Use Outcomes Among Homeless Clients with Serious Mental Illness: Comparing Housing First with Treatment First Programs. Community Ment Health J. 2011 Apr 1;47(2):227–32.

## Figures

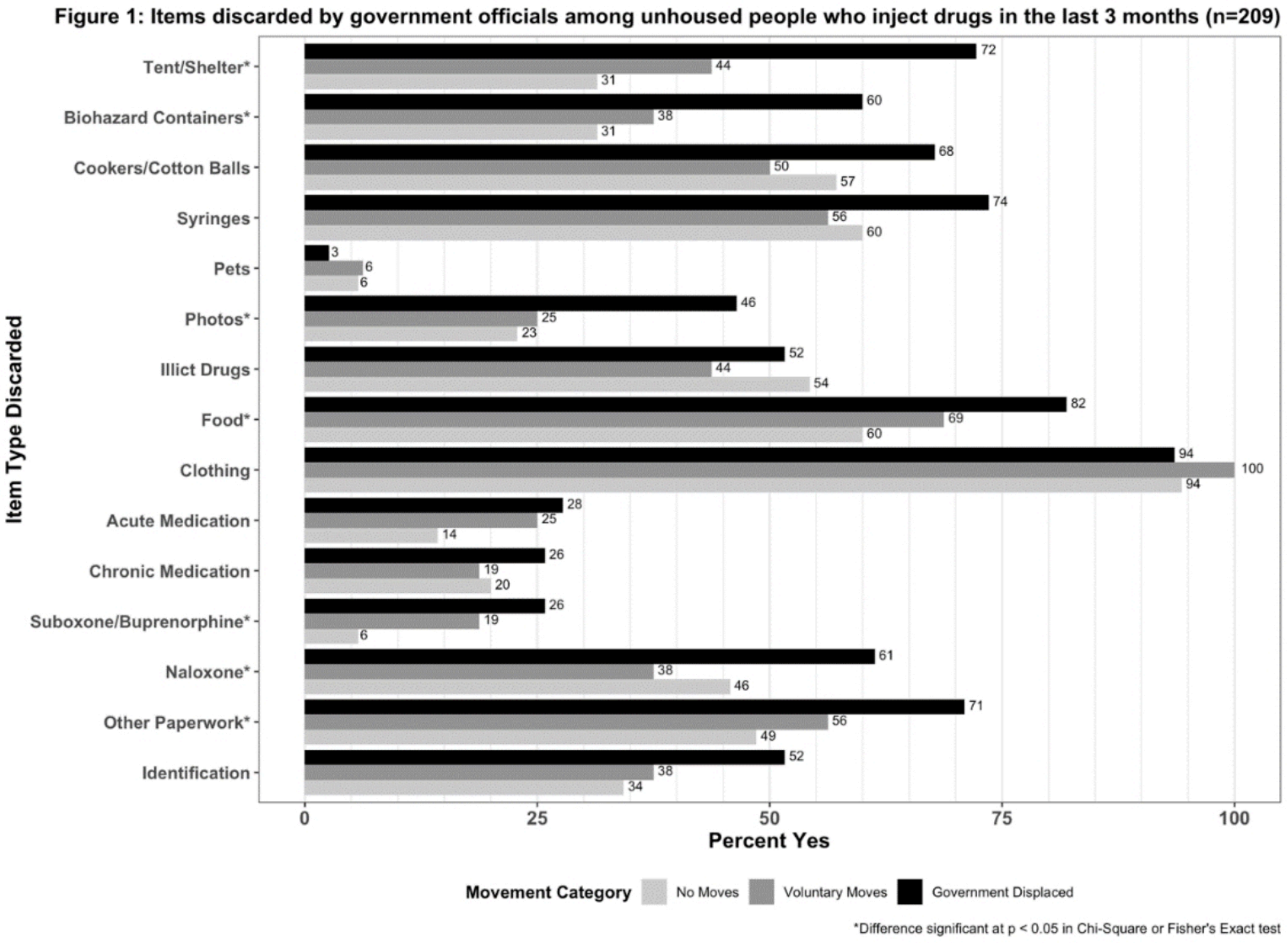

### Figure 1

See image above for figure legend
